# Supplementary material for: Causal interplay between lactose intolerance and gut microbiota: a combined bidirectional Mendelian randomization and in vivo validation study
Source: Front Nutr. 2026 Jun 1;13:1803337. doi: 10.3389/fnut.2026.1803337 (PMC13265576; doi:10.3389/fnut.2026.1803337)
Supplement: Supplementary file 1 [file Data_Sheet_1.zip › supplementary materials/Forward/leave one out cross validation/ebi-a-GCST90027587.finngen_R12_E4_LACTONAS.pdf]

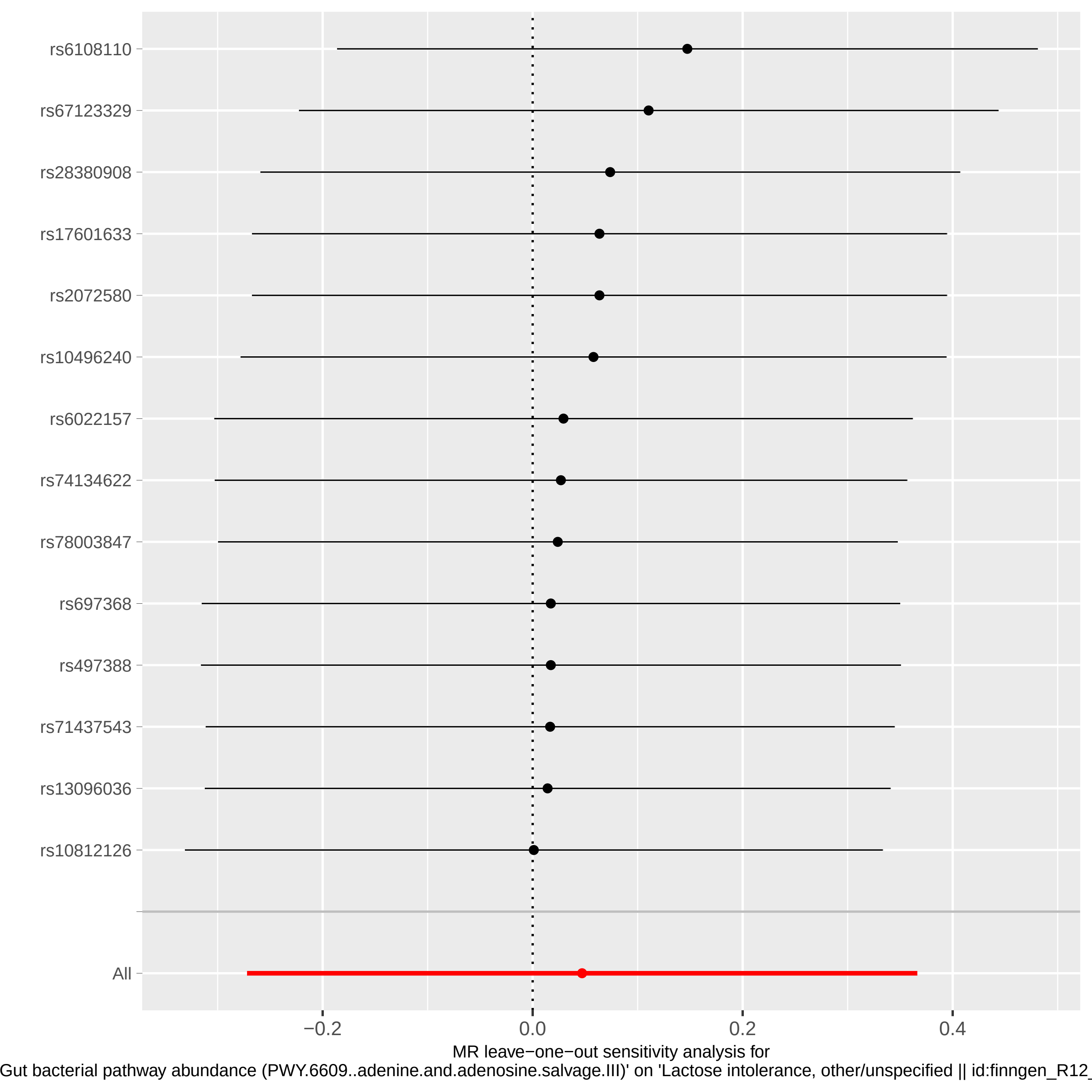

rs6108110
rs67123329
rs28380908
rs17601633
rs2072580
rs10496240
rs6022157
rs74134622
rs78003847
rs697368
rs497388
rs71437543
rs13096036
rs10812126
All
−0.2
0.0
0.2
0.4
MR leave−one−out sensitivity analysis for
Gut bacterial pathway abundance (PWY.6609..adenine.and.adenosine.salvage.III)' on 'Lactose intolerance, other/unspecified || id:finngen_R12_
